# Autologous Hematopoietic Stem Cell Transplantation for High-risk Acute Lymphoblastic Leukemia: non-Randomized Study with a maximum Follow-up of more than 22 Years

**DOI:** 10.4084/MJHID.2014.047

**Published:** 2014-07-01

**Authors:** Grzegorz Helbig, Malgorzata Krawczyk-Kulis, Malgorzata Kopera, Krystyna Jagoda, Patrycja Rzepka, Aleksandra Majewska-Tessar, Marta Hejla, Slawomira Kyrcz-Krzemien

**Affiliations:** 1 Department of Hematology and Bone Marrow Transplantation, Silesian Medical University, Katowice, Poland; 2 Students Research Group, Department of Hematology and Bone Marrow Transplantation, Silesian Medical University, Katowice, Poland

## Abstract

**Objective:**

To evaluate the efficacy and toxicity of autologous hematopoietic stem cell transplantation (AHSCT) for high-risk acute lymphoblastic leukemia (ALL).

**Material and methods:**

Overall, 128 high-risk ALL patients at a median age of 26 years (range 18–56 years) at diagnosis received AHSCT between 1991–2008. Induction treatment was anthracycline-based in all patients. Conditioning regimen consisted of CAV (cyclophosphamide, cytarabine, etoposide) in 125 patients whereas 3 subjects received cyclophosphamide and TBI (total body irradiation). Bone marrow was stored for 72 hours in 4°C and re-infused 24 hours after conditioning completion. Bone marrow was a source of stem cells in 119 patients, peripheral blood in 2 and 7 subjects received both bone marrow and peripheral blood.

**Results:**

With a median follow-up after AHSCT of 1.6 years (range 0.1–22.3 years), the probability of leukemia-free survival (LFS) for the whole group at 10 years was 27% and 23% at 20 years. Transplant-related mortality at 100 days after AHSCT was 3.2%. There was a strong tendency for better LFS for MRD-negative patients if compared with patients who had positive or unknown MRD status at AHSCT (32% vs 23% and 25%, respectively; p=0.06). There was no difference in LFS between B- and T-lineage ALL as well as between patients transplanted in first complete remission (CR1) and CR2. LFS at 10 years for patients with Philadelphia-positive (Ph+) ALL at transplant was 20% and this was comparable with subjects with negative and missing Ph status (26% and 28%; p=0.97).

**Conclusions:**

The results of AHSCT for high-risk ALL remains unsatisfactory with low probability of long-term LFS.

## Introduction

The clinical outcome of adult acute lymphoblastic leukemia (ALL) remains far unsatisfactory with a long-term survival of about 50% for patients <60 years and merely 10% for older subpopulation.^1^,^2^,^3^ An initial therapy including remission induction followed by an intensified consolidation results in complete remission (CR) rate of 80%–90% with early death in less than 10% of treated patients.^2^,^4^,^5^ The optimal post-remission therapy for adult ALL represents a major challenge and further management is usually risk-adapted,^6^ however the role of hematopoietic stem cell transplantation (HSCT) was found to be highly controversial as demonstrated by conflicting results of various studies. Some authors did not demonstrate any differences between chemotherapy and allogeneic/autologous HSCT^7^ whereas others reported on a significant superiority of allogeneic HSCT in high-risk ALL patients.^5^ A follow-up of the French LALA-87 study demonstrated a trend favoring an autologous HSCT (AHSCT) over chemotherapy in patients with high-risk features, but there was no difference between chemotherapy, autologous and allogeneic HSCT in standard-risk ALL population.^8^ The summary of the three randomized studies from LALA group comparing chemotherapy and AHSCT in high-risk ALL patients with no sibling donors found a significantly lower risk of relapse at 10 years in the autologous group with no improvement in overall survival.^9^ Recently published data indicates that minimal residual disease status (MRD) before AHSCT is a predictor of better clinical outcome in high-risk ALL subjects transplanted in first complete remission (CR1), but further prospective studies are required.^10^

The goal of our retrospective study was to analyze the results of AHSCT in 128 high-risk ALL patients.

## Material and Methods

### Study design

One hundred and twenty eight patients at a median age of 26 years (range 18–56 years) received AHSCT in our center in years 1991–2008. They were recruited from several hematologic institutions. Patients were eligible for AHSCT if they had no sibling or alternative matched donor and met the following criteria of high risk (HR) ALL (at least one criterion must be met): 1) age ≥ 35 years, 2) white blood cell (WBC) count at diagnosis ≥30×10^^9^/L for B-cell ALL and ≥100×10^^9^/L for T-cell ALL, 3) pro-B, early-T and mature T immunophenotype, 4) second or subsequent complete remission (CR) and 5) the presence of adverse cytogenetics: (Philadelphia-positive ALL, i.e. t(9;22) and/or BCR-ABL transcripts; ALL with 11q23 abnormality and/or MLL-AF4 transcripts; ALL with t(1;19) and/or E2A-PBX1 transcripts; ALL with complex karyotype and hypodiploidity). Due to the fact that some patients were referred for AHSCT from other centers, not all data were available for all patients.

### Induction treatment

Induction treatment was anthracycline-based in all study patients, but chemotherapy regimens varied depending on study protocol used. Most adults ALL patients received therapy according to the Polish Adult Leukemia Group (PALG): 4-91 (n=20); 4-96 (n=46); 4-2002 (n=28)^11^,^12^,^13^ or German Multicenter Study Group for Adult ALL (GMALL; n=34).^14^ Central nervous system (CNS) prophylaxis consisted of intrathecal administration of methotrexate, cytarabine and steroids and/or cranial irradiation (depending on study protocol and year of diagnosis). One hundred and seven patients achieved CR1 (84%) whereas 21 subjects had CR2 at transplant (16%).

### Investigation of minimal residual disease

Minimal residual disease (MRD) was evaluated in bone marrow samples on the day of aspiration by multiparametric flow cytometry: EPICS-XL (Beckman Coulter Inc. Marseille, France) and FACSCanto II (Becton Dickinson, Biosciences, San Jose, CA, USA). The “Stain&Lyse&Wash” method for staining of the surface markers and Fix&Perm Cell Permeabilization Kit (Invitrogen) for simultaneous staining of surface and intracellular markers were applied according to the producer’s instructions. At least 2–3 different aberrant phenotypes were examined to avoid false negative results. The isotypic negative controls and normal bone marrow samples were used to avoid false positive results. At diagnosis bone marrow samples were stained with a panel of 3–18 triple combinations of antigens. Only phenotypes that have been found to be informative for MRD examinations (>50% expression on leukemic cells before treatment and <0.1% in normal bone marrow) were used for disease monitoring. The 3 following aberrant phenotypes were analyzed: 1) ectopic phenotypes 2) asynchronous antigen expression/overexpression within the same line and 3) co-expression of antigens from different cell lines. The “quadrant” and “empty spaces” techniques were applied for searching of these abnormal phenotypes. MRD status was assessed before conditioning regimen using a panel of combinations of antibodies and level of 0.1% was used as a cut-off point.^14^ In sum, data on MRD were available for 57 patients; 40 were MRD negative and 17 MRD positive. The results of MRD were missing for 71 subjects.

All BCR-ABL positive patients had p190 transcripts and all they were found to have Philadelphia chromosome on cytogenetic study. BCR-ABL status was monitored using real-time polymerase chain reactions (PCR) assays.^15^ Data on Philadelphia status at transplant was known for 53 patients; and 5 out of them were Ph+ at transplant (9%). No patient received tyrosine kinase inhibitors, both before and after transplantation. No other cytogenetic high-risk patients were identified in our study group.

### Transplant details

The conditioning regimen consisted of CAV (cyclophosphamide 60mg/kg on days -3,-2; cytarabine 2000mg/m^2^ on days -3,-2,-1 and etoposide 800mg/m^2^ on days -3,-2) in 125 patients whereas 3 subjects received cyclophosphamide 120mg/kg on days -6,-5 and total body irradiation (TBI) with 12 Grey on days -3 to -1. Bone marrow was collected in general anesthesia, stored for 72 hours in 4°C without any processing and re-infused 24 hours after conditioning ending. Bone marrow was a source of stem cells in 119 patients, peripheral blood in 2 and 7 subjects received both bone marrow and peripheral blood. A median number of transplanted nuclear cells and CD34+ cells was 2.11×10^^8^/kg body weight (range 0.86–7.04) and 1.7×10^^6^/kg body weight (range 0.16–36.2), respectively. A median time to achieve an absolute neutrophil count >1.0×10^^9^/L and platelet count >50×10^^9^/L was 16 days. No granulocyte colony stimulating factor (G-CSF) was used. Patients demographic and transplant data were shown in table 1.

### Statistical analysis

The probability of leukemia-free survival (LFS) was defined as the time interval form transplant to the relapse or death in CR and it was calculated according to Kaplan-Meier method. All calculations were made from the date of transplantation. Comparisons between the variables were carried out by log-rank test. Statistical significance was defined at a P value <0.05. A Cox model was used to identify prognostic variables. The following variables were included in Cox regression model: gender, age, the type of induction protocol, WBC count at diagnosis, disease immunophenotype, CNS involvement, disease status at transplant, MRD status at transplant, year of transplant (before and after 2000), time from diagnosis to transplant, the number of transplanted NC and CD34+ cells. Transplant-related mortality (TRM) was defined as death within 100 days post autologous HSCT not related to the disease, relapse and progression.

## Results

**Figure 1:**
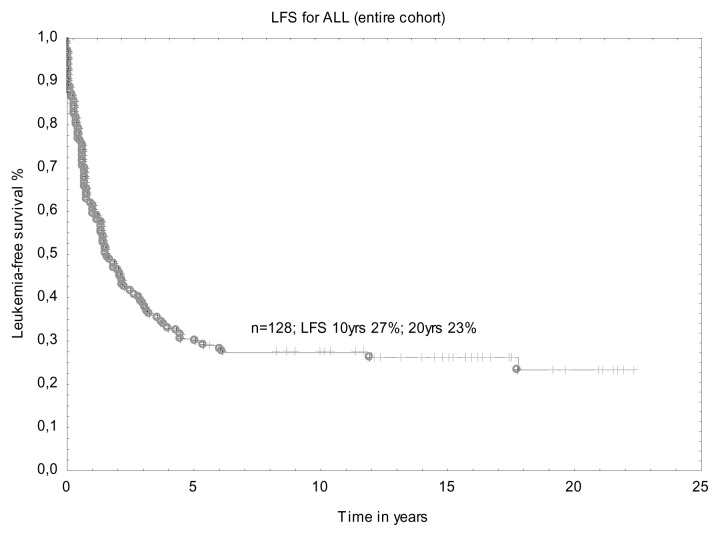
Leukemia-free survival after AHSCT (entire cohort).

One hundred and twenty eight patients at median age of 27 years at transplant (range 18.7 – 56.6 years) were analyzed in this retrospective study. A median time from diagnosis to transplant was 9.2 months (range 1.5–115.7 months). With a median follow-up after AHSCT of 1.6 years (range 0.1–22.3 years), the probability of LFS for the whole group at 10 years was 27% and 23% at 20 years (Figure 1). Four patients died due to infectious complications early after transplant and one died due to intracerebral hemorrhage (TRM=3.2%). The reason of death in the remaining subjects was disease relapse with subsequent resistance to re-induction treatment or therapy-related complications. One relapse occurred >10 years after transplant. Details of transplant-related complications were shown in table 2.

No difference in the probabilities of LFS was found between patients transplanted before (n=71) and after 2000 (n=57). No statistical difference was found between patients with B-cell and T-cell ALL as well as between patients transplanted in CR1 vs CR2 in regards to demographic and laboratory data. There was no difference in estimated LFS between B-lineage and T-lineage ALL at 10 years; 24% vs 34%, respectively (p=0.48) as well as for patients transplanted in CR1 and CR2 (30% vs 9%; p=0.22). There was a strong trend towards better LFS at 10 years for patients with negative MRD at transplant when compared with subjects who had positive or unknown MRD (32% vs 23% vs 25%; p=0.06). The impact of MRD status on LFS has not been also observed when we restricted our study cohort to the patients with known MRD status and CR1 at transplant; the probabilities of LFS at 10 years were 35% for MRD-negative and 30% for MRD-positive subjects, p=0.5. Only 5 patients were found to be Ph+ at transplant, but they remained MRD negative by flow cytometry. LFS at 10 years for this small subgroup was 20% and this was comparable with that seen for subjects with undetectable and missing Ph status (26% and 28%; p=0.97). No factor was found to have a significant impact on LFS in multivariate Cox regression model.

## Discussion

AHSCT for ALL has not shown a significant advantage over conventional chemotherapy in a majority of randomized studies and that was proved for any particular risk groups.^8^,^16^,^17^ It may result from two main aspects of AHSCT, namely 1) the contamination of graft by residual leukemic cells and 2) the lack of graft-versus-leukemia effect. There are only single reports on the use of different purging methods in patients undergoing AHSCT, but none of these studies demonstrated outcome benefits if compared with “no purging” procedure.^18^,^19^,^20^

Prior trials in high-risk ALL analyzed the efficacy of donor vs no donor outcomes after consolidation treatment. Then, the subjects with lacking donor were randomized between AHSCT and maintenance with chemotherapy. Unexpectedly, two large studies of EORTC and PETHEMA groups failed to prove that allogeneic sibling HSCT yielded a better LFS than AHSCT or chemotherapy. There was also no difference if AHSCT was compared with chemotherapy.^5^,^21^ The LALA-94 trial was the first that demonstrated the superiority of allogeneic HSCT in high risk ALL patients, but the same study did not show any advantage of AHSCT over chemotherapy.^2^ Moreover, the up-to-date largest international study showed that patients randomized to AHSCT had a significantly lower 5-year overall survival than these treated with chemotherapy (37% vs 46%).^22^ It should be emphasized that no beneficial effect of AHSCT in first complete remission was demonstrated if compared with chemotherapy in meta-analysis study with 2962 Philadelphia-negative ALL patients. The authors analyzed data from five randomized trials comparing AHSCT and chemotherapy and they found no difference in relapse rate. Moreover, there was a tendency toward higher TRM and inferior survival in autografted subjects. The conclusion from this analysis is that autograft has no advantage over chemotherapy in ALL transplanted in CR1.^16^ The results of our analysis despite many drawbacks resulting from a retrospective nature of the study, seem to be comparable with data provided by others. The LALA-85, -87 and -94 trials reported on 10-year probability of LFS of 20% for AHSCT in CR1 of high-risk ALL.^9^ It should be mentioned that the comparison with other studies is difficult, and it is probably due to the difference in conditioning regimen (usually TBI and Cyclophosphamide) and purging methods.^23^

The advantages of our study were a quite large number of included patients, a uniform conditioning regimen administered in most patients as well as the homogeneous use of bone marrow as a source of stem cells. However, the leukemic contamination was not examined in transplanted marrow. There were single attempts to improve the results of AHSCT in ALL setting by an intensification of conditioning regimen and the preemptive results based on the small number of included patients were found to be satisfactory. For transplanted subjects the LFS at 5 year was 53% with TRM of 5%.^24^

One may consider to offer AHSCT or allogeneic unrelated donor (URD) transplantation for high-risk ALL patients who lacking a family donor. A large study with 712 ALL patients compared the outcomes of autologous and allogeneic URD HSCT performed in first or second CR. There was no difference in engraftment rate, but TRM was significantly higher after URD transplantation (42%) than after autologous transplant (20%). Of note is that relapse rate was less frequently seen in the former group (14% in CR1 and 25% in CR2) if compared with the latter one (49% in CR1 and 64% in CR2). Nevertheless, overall survival rates at 3 years were comparable between URD HSCT and AHSCT for patients transplanted in CR1 (51% vs 44%) and CR2 (40% vs 32%).^25^

Some papers have shown that MRD status before transplant may have an impact on LFS in high risk adult patients.^10^,^26^ Patel et al. performed a sub-analysis including 25 ALL patients with known MRD status before AHSCT. It was demonstrated that the presence of MRD (≥10^−4^ ) was associated with lower LFS at 5 years (25%) if compared with MRD-negative (<10^−4^) subjects (77%).^10^ The other study included 123 high risk ALL patients autografted in CR1. The study patients were collected from eight European centers and MRD assessment was based on different methods (flow cytometry and polymerase chain reaction). The estimated 5-year LFS was significantly higher for subjects with negative MRD before transplant (57%) if compared with these transplanted with positive MRD (17%). That was also true for T-lineage ALL and a tendency was shown for B-origin ALL. In a multivariate analysis, only negative MRD was associated with better post-transplant outcome.^26^ Our study did not show a significant difference in LFS between MRD-positive and MRD-negative ALL patients at transplant. Moreover, the LFS was comparable between these two subgroups when the analysis was restricted to the patients undergoing AHSCT in CR1. However, our results should be interpreted with caution due to the retrospective nature of the study and limited number of included patients. The methodology of MRD assessment by flow cytometry has also changed during the study period. It should be highlighted that the prognostic value of MRD before transplant was statistically demonstrated only for transplantations performed using peripheral blood as a source of stem cells, but not for bone marrow.^26^

Another absorbing aspect of AHSCT relates to patients with Philadelphia-positive ALL that has never been considered as an option for this patient subgroup. One hundred and seventy seven Philadelphia-positive subjects were autografted and the results have recently been reported. It was demonstrated that year of transplantation was the only factor influencing the risk of treatment failure. The probability of LFS increased from 11% for transplants performed between 1996–2001, to 39% in years 2002–2006 and finally it reached 57% for years 2007–2010. The better results were shown for ALL patients treated with tyrosine kinase inhibitors (TKI): LFS at 3 years was 65%. The further studies are required to confirm these satisfactory results.^27^ The Ph status was known for 53 patients from our study cohort, and 9% of these subjects were Ph+ at transplant. None of these Ph+ patients received TKI and all were transplanted before 2000. The probability of 10-year LFS was 20% and this was comparable with Ph-negative and unknown subgroup. The other pre-TKI studies did not demonstrate any advantage of AHSCT over chemotherapy in this population.^22^,^28^ Conversely, several recently published studies have shown that AHSCT preceding by TKI and chemotherapy may result in similar outcome to that achieved by allogeneic HSCT.^29^,^30^ Based on the recent reports it seems reasonable to offer AHSCT for Ph+ ALL patients without a family donor and no detectable BCR-ABL transcript at transplant. However, there was lacking comparisons of matched URD HSCT with AHSCT in this cohort. In conclusion, AHSCT for high-risk ALL may constitute an alternative therapeutic option for patients who lacking a family/alternative donor, however the long-term results remain unsatisfactory. Prospective studies focusing on MRD status are required to re-evaluate the role of AHSCT for high-risk ALL.

## Figures and Tables

**Table 1:** Patients characteristics and transplant data.

Variable	B-cell ALL (n=93)	T-cell ALL (n=35)	p

Male/Female; no	57/36	26/9	0.21

Median age; years, range			
at diagnosis	26.2 (18–56)	25.4 (18.8–53.1)	0.93
at transplant	27.4 (18.7–56.6)	25.9 (19.4–53.5)	0.74

WBC count at diagnosis (x10^9/L); median, range	7.2 (0.5–524.0)	21.6 (2.1–622.0)	0.16

CNS involvement at diagnosis; no, %	5 (5)	1 (3)	0.53

Induction regimen; no, %			
GMALL	23 (25)	11 (31)	0.53
4-91	15 (16)	5 (14)	
4-96	35 (38)	11 (31)	
4-2002	20 (11)	8 (25)	

Median time from diagnosis to AHSCT; months, range	9.2 (1.5–115.7)	8.9 (2.7–53.7)	0.50

Hematologic status at AHSCT; no, %			
CR1	77 (83)	30 (85)	0.79
CR2	16 (17)	5 (15)	

BCR-ABL status at AHSCT; no, %			
Negative	42 (45)	6 (17)	0.53
Positive	5 (5)	0 (0)	
Missing	46 (50)	29 (83)	

MRD status at AHSCT; no, %			
Negative	28 (30)	12 (34)	0.25
Positive	15 (16)	2 (6)	
Missing	50 (54)	21 (60)	

NC count (10^^8^/kg);median, range	2.1 (0.9–7.0)	2.0 (0.8–4.8)	0.90

CD34+ count (10^^6^/L); median, range	1.6 (0.1–24.7)	2.2 (0.4–36.2)	0.30

ANC >1.0 (x10^^9^/L) post AHSCT (days) median, range	16 (12–28)	17 (11–45)	0.29

PLT >50 (x10^^9^/L) post AHSCT (days) median, range	15 (10–53)	16 (10–42)	0.52

Median time of AHSCT hospitalization; days, range	28 (18–180)	29 (19–52)	0.57

Median follow-up after AHSCT; years, range	1.63 (0.008–22.3)	1.49 (0.05–21.9)	0.49

Median follow-up from diagnosis; years, range	2.85 (0.54–26.2)	2.52 (0.56–26.4)	0.72

Legend: AHSCT=autologous hematopoietic stem cell transplantation; ALL=acute lymphoblastic leukemia; ANC=absolute neutrophil count; CNS= central nervous system; CR=complete remission; MRD=minimal residual disease; NC=nuclear cells; PLT=platelets

**Table 2:** Transplant-related complications.

Description of complication	Number of involved subjects; no, % (N=128)#
Fever of unknown origin	49 (38)
Stomatitis/pharyngitis	49 (38)
Any serious complaints	38 (30)
Herpetic stomatitis	11 (9)
Gastro-intestinal disturbances	11 (9)
Thrombosis and hemorrhage	10 (8)
Bronchitis/pneumonia	4 (3)
Urinary tract infection	4 (3)
Septic shock	3 (2)
Increased liver enzymes	3 (2)
Central catheter-related infections	3 (2)
Tonsillitis	1 (1)
Otitis	1 (1)

# single patient may have more than one complication.
